# Association between sedentary behavior and chronic kidney disease in Korean adults

**DOI:** 10.1186/s12889-022-14929-5

**Published:** 2023-02-10

**Authors:** Ye Seul Jang, Yu Shin Park, Hyunkyu Kim, Kyungduk Hurh, Eun-Cheol Park, Suk-Yong Jang

**Affiliations:** 1 Department of Public Health, Graduate School, Yonsei University, Seoul, Republic of Korea; 2 Institute of Health Services Research, Yonsei University, Seoul, Republic of Korea; 3 Department of Psychiatry, Yonsei University College of Medicine, Seoul, Republic of Korea; 4 Department of Preventive Medicine, Yonsei University College of Medicine, Seoul, Republic of Korea; 5 Department of Healthcare Management, Graduate School of Public Health, Yonsei University, 50 Yonsei-Ro, Seodaemun-Gu, Seoul, 03722 Republic of Korea

**Keywords:** Chronic kidney disease, Sedentary behavior, Albuminuria

## Abstract

**Background:**

Chronic kidney disease (CKD) is a significant health care burden, with a worldwide prevalence of approximately 11%. The general population spends over 50% of the awake time sedentary activities. However, to the best of our knowledge, no study has evaluated the association between sedentary time and CKD, with a focus on both kidney damage and kidney function, in the South Korean population. Accordingly, the present study aimed to address this gap in the knowledge.

**Method:**

We used data from the 8^th^ Korea National Health and Nutrition Examination Survey. The analysis included 9,534 participants, especially excluded those who had been diagnosed with kidney disease or who were currently undergoing treatment. Sedentary behavior was self-reported by the participants. An estimated glomerular filtration rate (eGFR) and/or albuminuria were used as measures for detection of CKD according to the guidelines of the Kidney Disease Improving Global Outcomes. We analyzed the data using multiple logistic regression.

**Results:**

Among the women, the risk of CKD was significantly greater among those who sat for ≥ 12 h/d relative to those who sat for < 6 h/d, after adjusting for physical activity and other covariates (odds ratio [OR]: 1.45, 95% confidence interval [CI]: 1.01–2.06). Similarly, among those who sat over 12 h/d, those who engaged in low levels of physical activity had a higher risk of CKD than those who engaged in high levels of activity (OR: 1.65, 95% CI: 1.04–2.61). No statistically significant results were found for men.

**Conclusion:**

Excessive sedentary behavior was associated with an increased risk of CKD, especially albuminuria, regardless of the level of physical activity, only in women. These findings emphasize the importance of avoiding excessive sitting for a long time and increasing overall physical activity levels.

## Introduction

The global prevalence of chronic kidney disease (CKD) is increasing, and it has become a serious public health problem worldwide [1]. In the United States, the reported prevalence of CKD among adults is 11.5%, and up to 40% among people aged ≥ 70 years [2]. CKD is characterized by progressive deterioration of kidney function. In addition, functional abnormalities are inferred by the glomerular filtration rate (GFR), whereas structural abnormalities are inferred from markers of kidney damage, including albuminuria. Both decreased GFR and albuminuria are independent risk factors for many manifestations of CKD, including stroke, heart failure, and coronary heart disease [3, 4]. According to a previous study, individuals with CKD have a higher risk of developing cardiovascular disease (CVD) than those with normal kidney function [5]. Therefore, since CKD shares many risk factors with CVD, including hypertension, diabetes, and obesity, it is important to identify the factors that contribute to the increased propensity for reduction in the eGFR and development of albuminuria.

It has been reported that the general population spends over 50% of the awake time engaging in sedentary activities [6]. The decrease in physical activity and continued increase in sedentary lifestyles owing to the development of transportation and the widespread availability of the Internet are gradually becoming global problems. According to the National Health and Nutrition Examination Survey(NHNES), in the USA, from 2003 to 2006, the average daily sitting time was 7.3–9.3 h, and, older people spent more time sitting than average [7]. In South Korea, it has been reported that adults spend > 7 h sitting down after waking up [8]. Sedentary time has a significant effect on health, and individuals who use more screen-based entertainment have a higher risk of developing cardiovascular diseases [9]. Furthermore, studies have reported that sedentary behavior is associated with cardiometabolic disease and mortality independent of physical activity [10]. A meta-analysis of nine prospective studies showed a nonlinear association between sedentary behavior and CVD events, with an increased risk associated with sitting > 10/d despite the presence of physical activity [10–13].

Similarly, previous studies demonstrated that prolonged sedentary behavior is associated with CKD development [14, 15], and most of the studies were conducted in Western countries [11, 16, 17], and to the best of our knowledge, no study has evaluated the association between sedentary time, kidney damage, and kidney function in the Korean population. According to a scoping review that self-reported sitting time in 29 countries in worldwide, Korea was the second-longest sedentary country among the countries participating in the study. In particular, the sitting time was longer than all Western countries [18]; Therefore, the purpose of this study was to investigate the association between time spent in sedentary behavior and the risk of CKD in the Korean population.

## Methods

### Data

Data were obtained from the 2019 and 2020 of the Korean National Health and Nutrition Examination Survey (KNHANES). The KNHANES is a cross-sectional nationwide survey conducted by the Korean Center for Disease Control and Prevention [19]. The survey is conducted in non-institutionalized Korean civilians throughout 192 regions to monitor trends in health risk factors and the prevalence of major chronic diseases, to evaluate the health and nutritional status of Koreans, and to provide relevant data for the development and evaluation of health policies and programs in Korea [19]. The KNHANES recruits a nationally representative sample of the South Korean population, using a complex and multistage clustered probability design.

### Participants

Since the variable of albuminuria was examined in the KNHANES from 2019 onwards, the current study used data from the 2019 and 2020 of the KNHANES. The data of 15,469 participants were examined. The exclusion criteria were aged < 19 years (*n* = 2,730), menstruation and pregnancy at the time of survey (*n* = 474), diagnosis of kidney disease or current treatment (*n* = 127), and missing data (*n* = 2,604). Finally, the study included 9,534 participants (4,491 men and 5,043 women).

### Variables

The main variable of interest was the participant’s sedentary behavior. The total weekday sedentary behavior was measured by the response to the following question, based on the format of the International Physical Activity Questionnaire [20]: “How much time do you typically spend sitting or lying down in a day?” Question about engagement with activities such as working at a desk or computer, reading books, writing, watching television, using the Internet, listening to music, etc. The participants’ responses were divided into the following four categories using quartiles [21]: 1^st^, < 6 h; 2^nd^, 6–8.9 h; 3^rd^, 9–11.9 h; 4^th^, ≥ 12 h/d).

The dependent variable was the prevalence of CKD. According to the Kidney Disease Improving Global Outcomes (KDIGO) guidelines, CKD is defined as the presence of moderately-to-severely decreased kidney function and/or kidney damage [22]. Reduced kidney function was identified by the eGFR, and kidney damage was identified by the presence of microalbuminuria [23]. The eGFR was calculated using the Modification of Diet in Renal Disease (MDRD) formula, Korean version, (eGFR < 60 mL/min/1.73 m^2^) [24]. Urinary albumin-creatinine ratio (UACR) of ≥ 17 in men or ≥ 25 mg/g in women indicated the presence of microalbuminuria [25].

The following covariates were included in the analyses: demographic factors (sex, age, educational level, and marital status), socioeconomic factors (region, occupation, and family income), and health-related factors (alcohol use and smoking). In addition, adjustments were made for the following variables: physical activity, which was divided into three categories using the metabolic equivalent of task (METS) [26], body mass index (BMI) was categorized into 3 groups according to the World Health Organization and Korean Society for the Study of Obesity standards [27], hypertension [28], and diabetes [29].

### Statistical analyses

Owing to sex differences in physical conditions, such as the urine albumin/creatinine ratio being high in women owing to low muscle mass and low levels of excretion of creatinine and urea, all analyses were stratified by sex. Descriptive analysis was performed to examine the distribution of the general characteristics of the study population by using chi-square test. Multiple logistic regression modelling was used to assess the association between sedentary behavior and CKD prevalence after adjusting for measured covariates in the study. Odds ratios (ORs) and 95% confidence intervals (CIs) were calculated to compare the subjects with CKD. SAS (version 9.4M6; SAS Institute Inc., Cary, NC, USA) was used for all statistical analyses.

## Result

Table 1 presents the general characteristics of the study population, stratified by sex. Of the 9,534 participants, 4,491 were men and 5,043 were women. Of these, 1,056 individuals, 581 men and 475 women, had CKD. The presence of CKD tended to increase with increasing age, reduction in family income, and increase in education level. Additionally, hypertension, diabetes, and obesity were associated with the prevalence of CKD.

**Table 1:** General characteristics of the study population

**Variables**	**Chronic Kidney Disease**													
	**Male**							**Female**						
	**Total**		**No**		**Yes**		***p-value***	**Total**		**No**		**Yes**		***p-value***
	**N**	**%**	**N**	**%**	**N**	**%**		**N**	**%**	**N**	**%**	**N**	**%**	
**Total (** ***N*** ** = 9,534)**	4,491	100.0	3,910	87.1	581	12.9		5,043	100.0	4,568	90.6	475	9.4	
**Sedentary Behavior**^**a**^							0.124							0.004
1st	1,107	24.6	944	85.3	163	14.7		1,326	26.3	1,224	92.3	102	7.7	
2nd	1,216	27.1	1,054	86.7	162	13.3		1,327	26.3	1,199	90.4	128	9.6	
3rd	1,073	23.9	947	88.3	126	11.7		1,274	25.3	1,162	91.2	112	8.8	
4th	1,095	24.4	965	88.1	130	11.9		1,116	22.1	983	88.1	133	11.9	
**Physical exercise(METS)**							0.001							< .0001
Low	2,111	47.0	1,801	85.3	310	14.7		2,718	53.9	2,418	89.0	300	11.0	
Moderate	1,748	38.9	1,534	87.8	214	12.2		1,957	38.8	1,797	91.8	160	8.2	
High	632	14.1	575	91.0	57	9.0		368	7.3	353	95.9	15	4.1	
**Age**							< .0001							< .0001
19–29	688	15.3	663	96.4	25	3.6		547	10.8	527	96.3	20	3.7	
30–39	659	14.7	630	95.6	29	4.4		654	13.0	626	95.7	28	4.3	
40–49	779	17.3	705	90.5	74	9.5		879	17.4	835	95.0	44	5.0	
50–59	839	18.7	723	86.2	116	13.8		1,094	21.7	1,015	92.8	79	7.2	
60–69	811	18.1	663	81.8	148	18.2		1,048	20.8	928	88.5	120	11.5	
70 ≤	715	15.9	526	73.6	189	26.4		821	16.3	637	77.6	184	22.4	
**Income**							< .0001							< .0001
Low	667	14.9	501	75.1	166	24.9		969	19.2	806	83.2	163	16.8	
Middle	2,340	52.1	2,054	87.8	286	12.2		2,598	51.5	2,380	91.6	218	8.4	
High	1,484	33.0	1,355	91.3	129	8.7		1,476	29.3	1,382	93.6	94	6.4	
**School**							< .0001							< .0001
Under middle school	905	20.2	690	76.2	215	23.8		1,625	32.2	1,349	83.0	276	17.0	
High school	1,673	37.3	1,475	88.2	198	11.8		1,653	32.8	1,535	92.9	118	7.1	
University and over	1,913	42.6	1,745	91.2	168	8.8		1,765	35.0	1,684	95.4	81	4.6	
**Region**							0.645							0.001
Urban	1,980	44.1	1,729	87.3	251	12.7		2,317	45.9	2,134	92.1	183	7.9	
Rural	2,511	55.9	2,181	86.9	330	13.1		2,726	54.1	2,434	89.3	292	10.7	
**Job**							< .0001							< .0001
White collar	1,303	29.0	1,206	92.6	97	7.4		1,109	22.0	1,056	95.2	53	4.8	
Pink collar	480	10.7	429	89.4	51	10.6		776	15.4	716	92.3	60	7.7	
Blue collar	1,456	32.4	1,239	85.1	217	14.9		771	15.3	684	88.7	87	11.3	
unemployed	1,252	27.9	1,036	82.7	216	17.3		2,387	47.3	2,112	88.5	275	11.5	
**Smoking**							0.041							0.136
Yes^b^	1,397	31.1	1,195	85.5	202	14.5		252	5.0	235	93.3	17	6.7	
No	3,094	68.9	2,715	87.8	379	12.2		4,791	95.0	4,333	90.4	458	9.6	
**Drinking**							0.038							< .0001
Yes	3,715	82.7	3,252	87.5	463	12.5		3,187	63.2	2,953	92.7	234	7.3	
No^c^	776	17.3	658	84.8	118	15.2		1,856	36.8	1,615	87.0	241	13.0	
**BMI**							0.001							< .0001
Underweight	104	2.3	93	89.4	11	10.6		233	4.6	225	96.6	8	3.4	
Normal	2,429	54.1	2,155	88.7	274	11.3		3,259	64.6	3,000	92.1	259	7.9	
Overweight	1,958	43.6	1,662	84.9	296	15.1		1,551	30.8	1,343	86.6	208	13.4	
**Hypertension**							< .0001							< .0001
No	1,517	33.8	1,445	95.3	72	4.7		2,398	47.6	2,309	96.3	89	3.7	
Pre-Hypertension	1,423	31.7	1,280	90.0	143	10.0		1,107	22.0	1,016	91.8	91	8.2	
Hypertension	1,551	34.5	1,185	76.4	366	23.6		1,538	30.5	1,243	80.8	295	19.2	
**Diabetes**							< .0001							< .0001
No	1,787	39.8	1,691	94.6	96	5.4		2,293	45.5	2,177	94.9	116	5.1	
Pre-Diabetes	1,931	43.0	1,702	88.1	229	11.9		2,078	41.2	1,883	90.6	195	9.4	
Diabetes	773	17.2	517	66.9	256	33.1		672	13.3	508	75.6	164	24.4	

^a^Using by Quartile = 1st: < 6 h,2nd: 6–8.9 h, 3rd: 9–11.9 h, 4th:12 h ≤

^b^eGFR < 60 mL/min/1.73 m2 or UACR of ≥ 17 mg/g for men or 25 ≥ mg/g for women

^c^Current smoker

^d^Not drinking at all in the last year, no lifetime experience

Table 2 shows the odds of developing CKD across different levels of sedentary behavior stratified by sex. Among women, long-term sedentary behavior (≥ 12 h/d) was significantly associated with the prevalence of CKD compared to short-term sedentary behavior (< 6 h/d), after adjusting for physical activity and other covariates (OR: 1.45, 95% CI: 1.01–2.06). No statistically significant associations were found among men.

**Table 2:** Association between CKD and subject demographic

**Variables**	**Male**					**Female**				
	**Chronic Kidney Disease**^**b**^				***p-value***	**Chronic Kidney Disease**^**b**^				***p-value***
	**OR**	**95% CI**				**OR**	**95% CI**			
**Sedentary Behavior**^**a**^										
1st	1.00					1.00				
2nd	1.08	(0.79	-	1.46)	0.64	1.30	(0.93	-	1.81)	0.12
3rd	0.80	(0.58	-	1.11)	0.18	1.29	(0.92	-	1.80)	0.14
4th	0.83	(0.60	-	1.15)	0.26	1.45	(1.01	-	2.06)	0.04
**Physical exercise(METS)**										
Low	1.22	(0.83	-	1.80)	0.81	2.17	(1.11	-	4.23)	0.11
Moderate	1.05	(0.71	-	1.56)	0.31	1.74	(0.88	-	3.45)	0.02
High	1.00					1.00				
**Age**										
19–29	1.00					1.00				
30–39	0.81	(0.41	-	1.60)	0.54	0.98	(0.50	-	1.94)	0.96
40–49	1.68	(0.92	-	3.09)	0.09	0.86	(0.48	-	1.56)	0.63
50–59	2.18	(1.18	-	4.03)	0.01	0.93	(0.48	-	1.78)	0.82
60–69	2.31	(1.33	-	4.01)	0.00	0.87	(0.43	-	1.74)	0.69
70 ≤	3.35	(1.85	-	6.04)	< .0001	1.42	(0.70	-	2.87)	0.33
**Income**										
Low	2.10	(1.43	-	3.08)	0.00	1.12	(0.76	-	1.65)	0.58
Middle	1.13	(0.85	-	1.49)	0.41	0.94	(0.69	-	1.30)	0.72
High	1.00					1.00				
**School**										
Under middle school	1.06	(0.73	-	1.53)	0.77	1.21	(0.75	-	1.94)	0.43
High school	1.06	(0.79	-	1.42)	0.70	1.25	(0.83	-	1.86)	0.28
University and over	1.00					1.00				
**Region**										
Urban	1.00					1.00				
Rural	1.00	(0.78	-	1.26)	0.97	1.17	(0.92	-	1.49)	0.20
**Job**										
White collar	1.00					1.00				
Pink collar	1.35	(0.84	-	2.15)	0.22	1.11	(0.66	-	1.88)	0.69
Blue collar	1.11	(0.77	-	1.60)	0.57	0.98	(0.61	-	1.59)	0.94
unemployed	1.14	(0.77	-	1.70)	0.51	1.12	(0.74	-	1.71)	0.59
**Smoking**										
Yes^c^	1.59	(1.26	-	2.02)	0.00	0.61	(0.34	-	1.10)	0.10
No	1.00					1.00				
**Drinking**										
Yes	1.25	(0.91	-	1.71)	0.17	0.87	(0.65	-	1.15)	0.32
No^d^	1.00					1.00				
**BMI**										
Underweight	1.48	(0.64	-	3.41)	0.36	0.73	(0.29	-	1.81)	0.49
Normal	1.00					1.00				
Overweight	1.39	(1.09	-	1.78)	0.01	1.28	(1.00	-	1.65)	0.05
**Hypertension**										
No	1.00					1.00				
Pre-Hypertension	1.52	(1.05		2.19)	0.03	2.03	(1.40		2.97)	0.00
Hypertension	2.64	(1.85	-	3.78)	< .0001	3.84	(2.61	-	5.65)	< .0001
**Diabetes**										
No	1.00					1.00				
Pre-Diabetes	1.62	(1.15		2.26)	0.01	1.05	(0.75		1.47)	0.77
Diabetes	4.96	(3.44	-	7.14)	< .0001	2.51	(1.71	-	3.68)	< .0001

^a^Using by Quartile = 1st: < 6 h,2nd: 6–8.9 h, 3rd: 9–11.9 h, 4th:12 h ≤

^b^CKD was identified eGFR < 60 mL/min/1.73 m2 or UACR of ≥ 17 mg/g for men or 25 ≥ mg/g for women

^c^Current smoker

^d^Not drinking at all in the last year, no lifetime experience

The results of the subgroup analysis are shown in Table 3. In the analyses stratified by independent variables, in the group with the highest levels of sedentary behavior, the participants with low physical activity showed a significantly increased risk of CKD (OR: 1.65, 95% CI: 1.04–2.61). A similar trend was observed for participants with hypertension. In addition, among the participants with high family income showed a sharp increase in the risk of CKD (OR: 3.37, 95% CI: 1.19–9.56).

**Table 3:** Results of subgroup analysis stratified by independent variables

	**Male**													**Female**												
	**Chronic Kidney Disease**^b^																									
	**Sedentary behavior**^a^													**Sedentary behavior**^a^												
	**1st**	**2nd**				**3rd**				**4th**				**1st**	**2nd**				**3rd**				**4th**			
	**OR**	**OR**	**95% CI**			**OR**	**95% CI**			**OR**	**95% CI**			**OR**	**OR**	**95% CI**			**OR**	**95% CI**			**OR**	**95% CI**		
**Physical exercise(METS)**																										
Low	1.00	1.33	(0.84	-	2.12)	1.09	(0.68	-	1.76)	1.02	(0.64	-	1.61)	1.00	1.13	(0.73	-	1.73)	1.42	(0.93	-	2.17)	1.65	(1.04	-	2.61)
Moderate	1.00	0.96	(0.60	-	1.55)	0.65	(0.37	-	1.17)	0.86	(0.49	-	1.51)	1.00	1.74	(1.03	-	2.94)	1.25	(0.69	-	2.24)	1.26	(0.70	-	2.25)
High	1.00	0.79	(0.34	-	1.80)	0.53	(0.21	-	1.37)	0.26	(0.05	-	1.27)	1.00	0.71	(0.10	-	5.05)	0.43	(0.07	-	2.51)	1.14	(0.16	-	7.96)
**Age**																										
19–29	1.00	0.56	(0.15	-	2.15)	0.92	(0.25	-	3.30)	0.56	(0.15	-	2.10)	1.00	0.27	(0.03	-	2.44)	0.30	(0.05	-	1.72)	0.46	(0.09	-	2.41)
30–39	1.00	0.78	(0.21	-	2.88)	0.81	(0.26	-	2.53)	0.39	(0.13	-	1.21)	1.00	0.84	(0.31	-	2.28)	0.38	(0.10	-	1.47)	0.58	(0.15	-	2.23)
40–49	1.00	1.07	(0.47	-	2.39)	0.55	(0.22	-	1.36)	1.06	(0.46	-	2.42)	1.00	2.84	(1.08	-	7.54)	1.34	(0.44	-	4.07)	2.20	(0.68	-	7.14)
50–59	1.00	1.67	(0.85	-	3.29)	0.93	(0.40	-	2.17)	0.82	(0.38	-	1.74)	1.00	1.02	(0.50	-	2.05)	1.51	(0.73	-	3.09)	1.23	(0.51	-	2.99)
60–69	1.00	1.22	(0.68	-	2.18)	0.88	(0.48	-	1.63)	0.80	(0.44	-	1.47)	1.00	1.41	(0.85	-	2.36)	1.15	(0.63	-	2.10)	1.57	(0.81	-	3.05)
70 ≤	1.00	0.69	(0.41	-	1.16)	0.62	(0.34	-	1.11)	0.95	(0.52	-	1.71)	1.00	0.98	(0.50	-	1.94)	1.60	(0.86	-	2.95)	1.32	(0.72	-	2.43)
**Hypertension**																										
No	1.00	1.24	(0.59	-	2.59)	1.11	(0.53	-	2.33)	0.58	(0.24	-	1.38)	1.00	1.70	(0.80	-	3.46)	1.48	(0.66	-	3.32)	2.08	(0.97	-	4.45)
Pre-Hypertension	1.00	0.61	(0.34	-	1.08)	0.56	(0.28	-	1.13)	0.66	(0.34	-	1.27)	1.00	1.36	(0.68	-	2.70)	0.98	(0.43	-	2.23)	0.97	(0.42	-	2.00)
Hypertension	1.00	1.42	(0.93	-	2.15)	0.91	(0.60	-	1.38)	1.03	(0.68	-	1.57)	1.00	1.06	(0.66	-	1.69)	1.38	(0.89	-	2.14)	1.41	(0.90	-	2.21)
**Diabetes**																										
No	1.00	0.83	(0.41	-	1.68)	1.27	(0.60	-	2.68)	0.54	(0.24	-	1.21)	1.00	1.52	(0.81	-	2.85)	1.18	(0.62	-	2.24)	1.40	(0.73	-	2.69)
Pre-Diabetes	1.00	0.82	(0.51	-	1.31)	0.54	(0.33	-	0.89)	0.71	(0.44	-	1.16)	1.00	1.22	(0.74	-	1.99)	0.99	(0.56	-	1.76)	1.67	(0.99	-	2.82)
Diabetes	1.00	1.79	(1.03	-	3.13)	1.00	(0.57	-	1.74)	1.32	(0.77	-	2.29)	1.00	1.17	(0.62	-	2.22)	1.62	(0.93	-	2.83)	1.02	(0.54	-	1.94)

^a^Using by Quartile = 1st: < 6 h,2nd: 6–8.9 h, 3rd: 9–11.9 h, 4th:12 h ≤

^b^CKD was identified eGFR < 60 mL/min/1.73 m2 or UACR of ≥ 17 mg/g for men or 25 ≥ mg/g for women

^*^All analysis was adjusted for all sociodemographic, economic, health-related factors considered in the study

Table 4 presents the association of each component of CKD, namely kidney function (estimated glomerular filtration rate) and kidney damage (microalbuminuria) with sedentary behavior, stratified by sex. After adjusting for potential covariates, the overall trend was similar to that observed in Table 2. The results pertaining to men showed no statistically significant association. Among women, those who sat for ≥ 12 h/d, regardless of physical activity level, had a higher risk of albuminuria relative to those who sat for < 6 h/d (OR: 1.45, 95% CI: 1.01–2.08). No statistically significant association was observed for kidney function.

**Table 4:** Association between sedentary behavior and each components of CKD definition

**Male**	**Chronic Kidney Disease**									
	**eGFR(< 60)**				***p*** **-value**	**Albuminuria(25 ≤)**				***p*** **-value**
	**OR**	**95% CI**				**OR**	**95% CI**			
**Sedentary Behavior**^**a**^										
1st	1.00					1.00				
2nd	4.84	(1.01	-	23.28)	0.049	1.04	(0.77	-	1.41)	0.11
3rd	2.65	(0.51	-	13.70)	0.24	0.78	(0.56	-	1.09)	0.16
4th	3.00	(0.63	-	14.19)	0.17	0.81	(0.58	-	1.12)	0.27
**Female**	**Chronic Kidney Disease**									
	**eGFR (< 60)**				***p*** **-value**	**Albuminuria(25 ≤)**				***p*** **-value**
	**OR**	**95% CI**				**OR**	**95% CI**			
**Sedentary Behavior**^**a**^										
1st	1.00					1.00				
2nd	1.92	(0.59	-	6.26)	0.277	1.30	(0.93	-	1.82)	0.124
3rd	2.51	(0.94	-	6.68)	0.07	1.23	(0.87	-	1.73)	0.23
4th	2.38	(0.69	-	8.18)	0.17	1.45	(1.01	-	2.08)	0.04

^a^Using by Quartile = 1st: < 6 h,2nd: 6–8.9 h, 3rd: 9–11.9 h, 4th:12 h ≤

^*^All analysis was adjusted for all sociodemographic, economic, health-related factors considered in the study

## Discussion

This nationwide population-based study found a positive association between long-term sedentary behavior (≥ 12 h/d) and increased risk of CKD, independent of physical activity level, BMI, hypertension, and other confounding variables. In addition, the participants with the low levels of physical activity tended to have an increased risk of CKD. Similarly, among the high physical activity individuals were tended to decreased risk of CKD as the level of sedentary behavior increased, but who sat over the ≥ 12 h/d still increased risk of CKD. Moreover, women with long sitting hours (≥ 12 h/d) had a 1.65-fold higher risk of microalbuminuria than those with short sitting hours (< 6 h/d).

Globally, the obesity epidemic and aging population have led to increasing health burdens of Acute kidney diseases and disorders (AKD) and CKD [30]. According to the World Health Organization, CKD has resulted in 1.2 million deaths and is the 12^th^ leading cause of death worldwide [31]. These results suggest the need for the detection, treatment, and evaluation of early stages of kidney disease to slow progression and prevent complications [32]. Previous studies have shown associations between sedentary behavior, physical activity, and kidney disease [16]. Some of the previous findings were consistent with our findings, especially that long sedentary behavior was associated with a high risk of CKD. Since few muscles are used in a sedentary lifestyle, it affects total blood volume and blood flow circulation, and as endothelial-dependent blood vessel relaxation capacity decreases, vascular endothelial damage due to blood flow resistance increases [33, 34]. Therefore, continued sedentary behavior could increase the risk of CKD owing to problems in blood flow circulation in the kidney or vascular structure [33–35].

Regular engagement with physical activity has been reported to have a positive effect on renal vascular resistance by reducing blood pressure and blood sugar levels, independent of other risk factors; the improvement in cardiovascular and endovascular functions through an improved insulin response has a positive effect on the vascular response of the kidney [35, 36]. Moderate and vigorous levels of physical activity are effective in improving kidney function by expanding the renal blood vessels [35]. This may explain our finding that individuals with low levels of physical activity and long periods of sedentary behavior had increased odds of developing CKD.

Our study extends the limited evidence base by suggesting that after adjusting for measured confounding variables, overall sedentary behavior is associated with CKD regardless of the physical activity measured by METS, only in women. According to previous study, they suggested that the higher levels of sedentary behavior, independent of physical activity, are associated with higher risk of CKD. Reasons for the observed gender difference in the relationship between sedentary behavior and CKD are not clear, although several hypotheses have been proposed. Previous report showed that a more positive association between time spent participating in sedentary behavior and pro-inflammatory biomarker levels, such as IL-6 and fibrinogen, in women compared to men [37]. The other study suggested that, although men report higher levels of sedentary behavior than women, men also tend to engage in different patterns of physical activity and that may protect against the effect of excess sedentary behavior [38]. Also, possible that sedentary behavior is associated with gender-specific differences in patterns of other potentially deleterious health behavior such as snacking [39]. Gender-specific differences in the accuracy of self-reported sitting time also potentially could act to dilute the strength of the measured associations in men [40]. Due to the inconsistency between studies evaluating the sex-specific association between sedentary behavior and CKD, more studies are needed to clarify this.

Both eGFR and albuminuria reflect the excretory function of the glomerulus and are the effective measures of kidney disease [32]. Through subgroup analysis, we found that the highest level of sedentary behavior was independently associated with kidney damage, regardless of the level of physical activity engaged in. From a pathophysiological perspective, albuminuria has been used as a biomarker of generalized endothelial dysfunction and capillary dilution [41, 42]. Therefore, our results show that sedentary lifestyle might contribute to the development of generalized endothelial dysfunction and capillary dilution, especially in women. These results are consistent with those of a previous study [23].

A decrease in the eGFR is the first step in the development of kidney failure and a meaningful indicator of CKD [30]. Most previous studies have shown associations between sedentary behavior and eGFR [16, 33]. However, in our study, we cannot find the statistically association. Presumably due to differences in self-reported measurements of physical activity and sedentary behavior or differences in sample size and study population.

Although this study showed that sedentary behavior is associated with the risk of CKD, it has some limitations. First, this study used a cross-sectional data set; thus, we could only determine the association and should beware to investigate the causal relationship between the variables. According to another study, reduced renal function was associated with a significant increase in sitting time [43]. Therefore, a similar observation is likely to be derived in the reverse direction. However, in our study, we attempted to minimize it by excluding those who were diagnosed with kidney disease or who were currently receiving treatment. Because, by physicians, they could affect health behavioral pattern, such as more exercise or reduce sitting time or medication use that can affect kidney disease. However, additional research is needed to accurately infer causality. Second, since the level of sedentary behavior was self-reported, the effect of recall bias could not be eliminated, and the responses might not have been accurate. Third, as we excluded unavailable missing data which accounted for nearly 15% of the original survey, we might have issues with representativeness. In hence, further research should be considered to make up for the missing data. Fourth, eGFR calculated using MDRD formula, but this formulas have some limitations [44]. So other variables that are accurately estimate kidney function, such as cystatin C, should be considered in future studies. Finally, although we adjusted for covariates related to sedentary behavior and chronic kidney disease, the confounding effect cannot be completely excluded because other potential confounding variables might exist such as unhealthy diet or other medication use.

Despite these limitations, this study has several strengths. First, since this study included a nationally representative sample, the results can be generalized to the Korean population. Second, to the best of our knowledge this study was the first to find a positive association between sedentary behavior and CKD, with a focus on both kidney function and kidney damage, in Korean adults.

## Conclusion

This study provides the additional evidence by showing that those who spent ≥ 12 h/d in sedentary behavior were at a high risk of developing CKD, only in women, regardless of the level of physical activity they engaged in and other confounding variables. The other components of CKD showed similar associations with sedentary lifestyle, especially when kidney damage was defined on the basis of microalbuminuria. In addition, the association between sedentary behavior and the risk of CKD was modified by the level of physical activity, as measured by METS, where in the group with long-term sedentary behavior (≥ 12 h/d), the risk of CKD was significantly higher in the low-level exercise subgroup than in the high-level exercise group. These findings emphasize the importance of avoiding sitting for a long time and increasing the levels of physical activity.

## Data Availability

Data used in this study was from 2019,2020 KNHANES, Raw data as a whole is available to the public, and data can be downloaded from the KNHANES official website (https://knhanes.kdca.go.kr/).
